# Genetics of SLE: Functional Relevance for Monocytes/Macrophages in Disease

**DOI:** 10.1155/2012/582352

**Published:** 2012-10-16

**Authors:** Jennifer C. Byrne, Joan Ní Gabhann, Elisa Lazzari, Rebecca Mahony, Siobhán Smith, Kevin Stacey, Claire Wynne, Caroline A. Jefferies

**Affiliations:** Molecular and Cellular Therapeutics and RCSI Research Institute, Royal College of Surgeons in Ireland, Dublin 2, Ireland

## Abstract

Genetic studies in the last 5 years have greatly facilitated our understanding of how the dysregulation of diverse components of the innate immune system contributes to pathophysiology of SLE. A role for macrophages in the pathogenesis of SLE was first proposed as early as the 1980s following the discovery that SLE macrophages were defective in their ability to clear apoptotic cell debris, thus prolonging exposure of potential autoantigens to the adaptive immune response. More recently, there is an emerging appreciation of the contribution both monocytes and macrophages play in orchestrating immune responses with perturbations in their activation or regulation leading to immune dysregulation. This paper will focus on understanding the relevance of genes identified as being associated with innate immune function of monocytes and macrophages and development of SLE, particularly with respect to their role in (1) immune complex (IC) recognition and clearance, (2) nucleic acid recognition via toll-like receptors (TLRs) and downstream signalling, and (3) interferon signalling. Particular attention will be paid to the functional consequences these genetic associations have for disease susceptibility or pathogenesis.

## 1. Macrophages in Disease: SLE Candidate Genes and Functional Relevance

**Figure 1:**
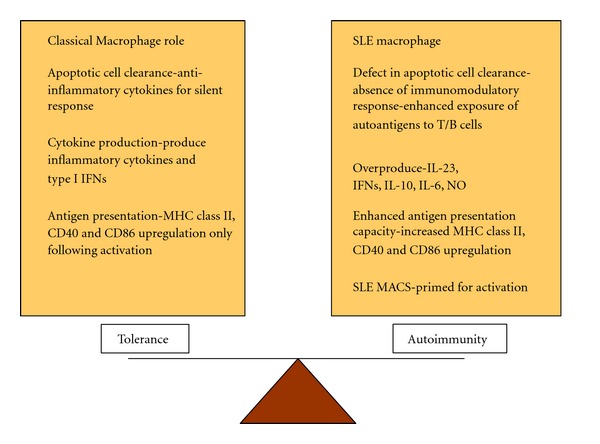
Dysregulation of macrophage function in SLE. The ability of the immune system to regulate macrophage function is altered in patients suffering from SLE. SLE macrophages have a defect in apoptotic cell clearance, overproduce IL-21, IFNs, IL-10, IL-6, and NO, have enhanced antigen presentation capacity and are primed for activation, leading to a skew towards autoimmunity.

Systemic lupus erythematosus (SLE) is a multisystem chronic autoimmune disease, which affects approximately 0.1% of the population, with women being approximately nine times more likely to develop the disease than men [1]. SLE is a complex disease encompassing a broad spectrum of clinical symptoms, particular combinations of which can result in varying disease severity. To date the majority of work undertaken with respect to understanding the pathophysiology of this condition has focused on the autoreactive B and T lymphocytes [2]. However, recently attention has shifted to the role of the innate immune system and particularly myeloid cells in disease. Both monocytes and macrophages are phenotypically altered in SLE, with SLE macrophages demonstrated to have reduced uptake of apoptotic cells, enhanced activatory status, an altered skew of proinflammatory and anti-inflammatory macrophages and an overproduction of inflammatory cytokines such as tumour necrosis factor-alpha (TNF-*α*), interleukin-6 (IL-6), interleukin-10 (IL-10), and antiviral type I interferons (IFNs) (Figure 1) [3–5]. As such, SLE monocytes and macrophages present self-antigens to autoreactive T cells in an inflammatory context, rather than the immuno-silent presentation normally associated with material from apoptotic cells [3]. In addition to this, the overproduction of type I IFNs by myeloid cells (including dendritic cells) also contributes to the breaking of immune tolerance due to their ability to stimulate antibody production and class switching from B cells [4]. The inadequate regulation of these processes in myeloid cells may be as a result of the influence of variants within SLE susceptibility genes. 

 Genetic analysis in human and murine studies indicate that susceptibility to SLE is heritable and that a number of different genetic loci are associated with disease risk [5]. Both candidate gene studies and Genome-wide association studies (GWAS) have unearthed many genes whose function can be clustered into 3 different categories, each clearly rooted in innate immune cell signalling and function (Table 1): (1) immune complex (IC) recognition and clearance such as the complement components and the Fc gamma receptors [6–8]; (2) nucleic acid recognition via toll-like receptors (TLRs) [9–11] and downstream signalling components such as TNF receptor-associated factor-6 (TRAF6) [12] and interferon regulatory factors (IRFs) [13] and (3) interferon signalling [14]. Characterisation of the molecular involvement of many of these genes in the function of SLE monocytes and macrophages has placed these cells as key orchestrators of SLE pathogenesis. Whilst the focus of this paper is the involvement of these candidate genes in macrophage function and their contribution to SLE pathology, it must be stressed that many of the candidate genes discussed below, particularly those that regulate type I IFN production, also play an important role in dendritic cell-driven autoimmune pathology [15, 16].

## 2. Immune Complex Recognition and Uptake

### 2.1. The Complement System and Its Association with SLE

The principal function of activated components of the complement system include production of inflammatory and chemotactic proteins (C3a and C5a), cell lysis through the formation of the membrane attack complex (complex of C5b-9 proteins), and most importantly in the context of SLE, recognition and clearance of immune complexes and apoptotic cells (C1–C4) [6–8, 44]. Although genetic deficiencies in individual loci are rare, homozygous deficiency of each of the classical pathway components (C1q, C1r, C1s, C4, and C2) has been shown to be associated with SLE in humans [22]. A hierarchy of susceptibility and severity of disease is present where association is greatest with homozygous C1q deficiency followed by homozygous C4 and C2 deficiency [8]. Hereditary deficiencies of C1s and C1r are rarer than that of C1q and, in the majority of cases, deficiencies of both these components are inherited together [19, 20]. Both C1q and C4 are important in clearance of apoptotic cells and immune complexes, thereby preventing inappropriate activation of autoreactive B and T cells. Thus, reduced functioning of this important housekeeping function of complement proteins is strongly associated with increased risk of developing SLE [13–15, 19, 45].

C1q functions in facilitating clearance of immune complexes and apoptotic cells, thus protecting against autoimmunity. In addition, recent work has demonstrated that C1q can protect against SLE by preventing the production of type I IFN by dendritic cells [45, 46]. Individuals with a congenital deficiency of *C1q* gene (C1qD) develop SLE-like symptoms at more than 90% prevalence [47, 48]. Interestingly, the importance of ethnicity and the possible influence of haplotypes is highlighted by the observation that although C1q deficiency has been reported in Turkish [17] and Mexican [18] individuals affected by SLE, no association has been in Malaysian patients [49]. 

Homozygous deficiency of complement C4 is one of the strongest genetic risk factors for SLE and results in lupus-like disease in approximately 80% of the 28 known affected individuals [23, 24, 47]. To date, 28 individuals with complete C4 deficiency from 19 families have been reported, among these 15 individuals developed SLE, 7 developed lupus-like disease and four of the remaining subjects had kidney disease [24]. Through a five nucleotide substitution in exon 26, the *C4 *gene can encode either a C4A or a C4B protein [50], both of which have differential functions. C4A preferentially binds to amino groups in immune complexes and is the preferential ligand for complement receptor 1 (CR1) [51] whereas C4B is thought to be a more potent initiator of the complement activation cascade. The complement *C4* gene located in the class III region of the major histocompatibility complex (MHC) on chromosome 6p21.3 and exhibits significant interindividual copy number variation (CNV). Boteva et al. demonstrated low *C4A* genome copy number significantly predisposed to SLE in UK and Spanish populations (*P* < 0.001) however, high *C4A* genome copy number was not associated with disease in either case (*P* = 0.63 and *P* = 0.76, resp.) [52]. Interestingly, *C4B* genome copy number demonstrated no association in the UK SLE group but was significantly associated with the Spanish cohort (*P* = 0.001). The discrepancies reported across different patient populations with respect to *C4* copy number suggests that partial *C4* deficiency states secondary to low *C4A* or *C4B* copy number are not independent genetic risk factors for susceptibility to disease [52, 53]. 

In addition to the rare inherited immunodeficiencies observed, many SLE patients have reduced levels of circulating C1q or C4 as a result of autoantibodies against these proteins, thus resulting in loss of their protective functions. Thus combined, mutations or decreased function of the early complement components has a profound effect on an individual's susceptibility to developing SLE.

### 2.2. Fc Gamma Receptors

Studies have investigated the contribution of the Fc-gamma family of receptors (Fc*γ*Rs) to the pathogenesis of SLE given their role in the recognition of the Fc portion of IgG and subsequent responses to circulating and deposited immune complexes. Recent work in animal models indicates that the development of many human autoimmune diseases might be caused by impairment of the Fc*γ*R regulatory system (reviewed in [54]). Fc*γ*Rs bind IgG, and can be further classified as activatory (Fc*γ*RI, IIA, IIIA, IIIB, and IV) or inhibitory (Fc*γ*RIIB) following IgG binding [55]. Additionally they can be subcategorised by relative affinity for IgG, with Fc**γ**RI having highest affinity, while Fc*γ*RII and III display lower affinity [56]. Currently there are no known polymorphisms in the *Fc*γ*RI* gene reported in humans and the rare individuals lacking this gene are healthy with no signs of autoimmune immune pathology [57]. However, polymorphisms in the activatory receptors *Fc*γ*RIIA* and *Fc*γ*RIIIA* have been identified [58–64].

Fc*γ*RII and III are encoded by two families of genes (*FCGR2, FCGR3*) clustered on chromosome 1q23-24, each containing multiple distinct genes [58]. Fc*γ*RIIA is a low-affinity receptor, comprised of multiple isoforms, which is expressed by B cells, monocytes, macrophages, and dendritic cells (DCs). It has two codominantly expressed alleles, R131 and H131, which differ in their affinity for IgG subclasses. Substitition of arginine to histidine at position 131 at the membrane proximal portion of the receptor results in enhanced affinity of Fc*γ*RIIA for binding of IgG2 and IgG3 by the H131 variant and increased levels of phagocytosis [59]. The allelic variant of Fc*γ*RIIA (R131) has been found to be strongly associated with lupus nephritis and renal failure in Brazilian lupus patients (*P* = 0.06) [60]. Interestingly meta analysis of European, African, and Asian populations demonstrated a significant association between the homozygous RR genotype and the development of SLE (*P* = 0.0016). This polymorphism was shown to increase the risk of devolving SLE 1.3-fold [61]. However analysis of this polymorphism in a Malaysian population found no significant association with disease [62]. 

*Fc*γ*RIII* encodes an activatory Fc*γ*R which is expressed on NK cells and monocytes, and has two isoforms: Fc*γ*RIIIA and Fc*γ*RIIIB. The wild-type sequence at position 176 encodes a phenylalanine (176-F) while the polymorphic variant is 176-valine (176-V) resulting in increased binding of IgG1 and IgG3 [63]. Recent studies in Japanese and Chinese patient cohorts found that positivity for the 176F allele was significantly increased in patients (*P* = 0.02 and *P* = 0.05 resp.), indicating a significant association of this allele with SLE [64, 65]. Additionally a significant association with this polymorphism and the development of lupus nephritis was observed among the Japanese patient cohort (*P* = 0.03) [64]. 

Fc*γ*RIIIB is an alternative membrane form of Fc*γ*RIII that is predominantly expressed on neutrophils and preferentially binds IgG1 and IgG3. The *Fc*γ*RIIIB *gene has three polymorphic forms known as HNA-1a, HNA-1b, and HNA-1c, encoded by the alleles FCGR3B*01, FCGR3B*02, and FCGR3B*03 (also referred to NA1, NA2, and SH) [66]. These different isoforms of *Fc*γ*RIII* exhibit differential function with increased levels of phagocytosis reported for FCGR3B*01 homozygotes compared to cells from FCGR3B*02 homozygotes, despite similar levels of receptor expression [67]. Reduced function of the FCGR3B*02 allele has been associated with impaired IC clearance in Caucasian populations [68] and has been strongly associated with disease susceptibility in Japanese and Thai populations (*P* = 0.008 and *P* = 0.02, resp.) [69, 70] and significantly associated with the development of lupus nephritis among the Japanese patient cohort (*P* = 0.007), whereas as no association was found in other population studies [62, 71]. 

As an inhibitory Fc*γ*R, loss of Fc*γ*RIIB not surprisingly results in development of lupus-like symptoms in mice, with the development of autoantibodies and autoimmune glomerulonephritis, consistent with a lack of inhibitory mechanisms on the development of autoreactive B cells [72]. Subsequent studies have demonstrated that increasing the expression of Fc*γ*RIIB in B cells derived from autoimmune-prone mice restored tolerance and prevented autoimmune disease [73]. With respect to the role of Fc*γ*RIIB in human autoimmune disease, reduced expression of Fc*γ*RIIb has been reported for memory B cells and plasma cells from SLE patients [74]. Interestingly, a polymorphism of Fc*γ*RIIb which changes the threonine at position 232 to an isoleucine (I232T) was found to be associated with SLE as positivity for the 232I allele was significantly decreased in SLE patients suggesting a significant association of the 232T/T genotype with SLE [64]. This study also found that the odds ratios (ORs) for the development of SLE among individuals with the T/T and I/T genotypes versus the I/I genotype were 2.3 and 1.1, respectively. A further comparison of genotype frequencies with patient clinical data revealed that *FCGR2B *polymorphismsstrongly associated with lupus nephritis (*P* = 0.01). This amino acid is in the transmembrane domain of Fc*γ*RIIb, and the polymorphism reduces the signalling capability of Fc*γ*RIIb due to its exclusion from lipid rafts [75]. Thus balanced signalling through activatory and inhibitory Fc*γ*Rs regulates the activity of various cells in the immune system and genetic evidence in both mice and humans strongly supports the role of this receptor family in preventing the development of autoimmunity.

### 2.3. CD11b/ITGAM

*ITGAM* encodes integrin alpha-M (also commonly known as CD11b or complement receptor 3), the alpha chain of *α*M*β*2 integrin which binds the cleavage fragment of complement component C3b, an opsonin and facilitates uptake of C3b-coated particles/pathogens into phagocytic cells (reviewed in [76]). Genetic association of ITGAM with SLE was found independently in 2 European GWAS [77, 78], with a non-synonomous functional variant being identified in a subsequent study [79]. Functionally this variant encodes an arginine to histine mutation at amino acid 77 which alters both the structure and function of integrin *α*M, thus reducing its ability to clear immune complexes [80]. 

## 3. Toll-Like Receptor Signalling and IFN Induction in SLE

Our increased awareness of the role played by cells of the innate immune system in disease has stemmed from the discovery of families of innate immune receptors, such as the TLRs, which have evolved to recognise and discriminate between different classes of pathogenesis reviewed in [81]. A link between antiviral pathogen recognition receptors and SLE is now well established, thus giving credence perhaps to the long-held view point that viral infection plays an important role in either the etiology of SLE or in driving flares in affected individuals [82]. With respect to SLE, receptors that can recognise viral nucleic acids, such as the endosomally located antiviral TLRs (TLR3, 7/8, and 9) [9–11] the intracellular RIG-I-like receptors (RLRs) [83] and AIM2-like receptors (ALRs) receptor families [84], have been implicated in SLE. There now exists strong genetic and functional evidence that RNA/DNA immune-complexes found in lupus patients can drive IFN-*α* production through the activation of TLR7 or TLR9 [85], respectively, indicating that TLR7/9 activation may be an important primary trigger for the generation of autoimmune disease (reviewed in [5, 86]). Plasmacytoid dendritic cells have been identified as the primary interferon-producing cells [87], however immature monocytes have also been demonstrated to produce significant levels of IFN-*α* in a mouse model of lupus and also in human SLE monocytes in response to immune complex activation [88, 89]. In addition to the viral TLRs themselves playing a role in the pathogenesis of this condition, downstream signalling components of these and their products may also contribute to the progression of this condition. Firstly, it is well documented that roughly half of all SLE patients overexpress IFN-*α*, thus giving rise to changes of gene expression that can be detected in peripheral blood monocytes, termed the IFN gene signature [90–93]. More recently, the activity and expression of certain members of the IRF family of transcription factors which regulate IFN production and mediate its effects, specifically IRF3 and IRF5, have been shown to be enhanced in SLE monocytes, resulting in increased expression of a subset of IRF-dependent genes [89, 94, 95]. For example, recent studies have shown that the levels of IRF3 bound to the promoter of a key pathogenic cytokine in SLE, IL-23, are enhanced in monocytes from SLE patients, thus resulting in increased basal production of this cytokine in SLE monocytes [95]. Likewise, monocytes from SLE patients present increased basal levels of nuclear IRF5 thus potentially contributing to enhanced production of the cytokines IFN-*α*, TNF-*α*, and IL-6 [96]. Thus not only are the triggers for activating SLE monocytes or macrophages in abundance due to impaired apoptotic cell clearance but also key downstream transcription factors such as the IRF family appear to be hyperresponsive in SLE monocytes [96], a finding inspired by genetic evidence linking IRF5 to disease [97]. 

### 3.1. Genetic Association of Antiviral Toll-Like Receptors TLR7 and TLR9 with SLE

With respect to the initial recognition of self-RNA and self-DNA by the antiviral TLRs, there have been several genetic studies in both human and murine models that further implicate these receptors in the pathogenesis of this condition in particular TLRs 7 and 9 [25–27, 88–96, 98–102].

#### 3.1.1. Toll Like Receptor 7 (TLR7)

Mice lacking the *TLR7* gene (located at Xp22.2) exhibit ameliorated disease, decreased lymphocyte activation and a marked reduction in the levels of RNA-containing antigens [98]. Interestingly, BBXSB/MpJ (BXSB) mice bearing the *Yaa* gene (Y chromosome-linked autoimmune acceleration gene) spontaneously develop a lupus-like autoimmunity, with males being affected much earlier and to a greater extent than their female counterparts. These *Yaa* containing mice were found to have increased expression of TLR7 due to the translocation of approximately 17 genes, including TLR7, onto the pseudoautosomal region of the Y chromosome [99, 100]. Deane et al. (2007) demonstrated that this duplication of the *TLR7* gene and as a result, increased TLR7 expression, promoted the production of RNA-containing autoantibodies and development of lupus nephritis [101]. Although murine studies have indicated associations between *TLR7* gene variations and SLE, there is controversy regarding human association studies. Using candidate gene approaches, Shen et al. (2010) investigated a role for TLR7 in SLE in Eastern Asian populations in which they identified a functional polymorphism in 3′ UTR of the *TLR7* gene. This common variant (rs3853839G/C) was found to be robustly associated with SLE (*P* = 0.016), with a stronger effect seen in male subjects compared to their female counterparts [25]. The elevated levels of *TLR7* transcripts and as a result, the enhanced IFN signature in patients with the G-allele of this single nucleotide polymorphism (SNP), have supported a functional role for this polymorphism in SLE. However when this SNP was studied in a non-Asian population, there was no evidence for this SNP as a risk factor for SLE in males with only females of non-Asian descent showing this association [102]. Following on from this multicentre study, Kawasaki et al. (2011), observed two additional variants located within the intron (rs179019A/C and rs179010T/C) that were also associated with SLE in a Japanese cohort (*P* = 0.016 and 0.018, resp.) thus further supporting the role of TLR7 as a risk factor for the development of this autoimmune condition [26]. Further studies into TLR7 polymorphisms in a Brazilian population also suggested the *TLR7* SNP rs179008A/T as an SLE susceptibility factor in women of European descent (*P* = 0.020); however, this was not replicated in a Spanish population [27, 103]. Moreover an additional study into the role of copy number variants of *TLR7* in SLE identified that increased *TLR7* copy number was also a risk factor for the onset of juvenile SLE [104].

#### 3.1.2. Toll-Like Receptor 9 (TLR9)

In addition to enhanced TLR7 expression, TLR9 has also been demonstrated to be upregulated in SLE B cells [105], further implicating a role for these viral TLRs in B cell tolerance and as result in the progression of SLE. In murine models, a role for TLR9 in disease susceptibility has also been examined with varying results. Christensen et al. (2005) demonstrated that TLR9 knockout mice crossed with lupus-prone mice exhibit decreased levels of anti-DNA antibodies implicating this gene as important in the progression of this condition [11]. However, in contrast the genetic ablation study carried out by Wu and Peng investigating the role of TLR9 in SLE, demonstrated that MRL mice lacking TLR9 developed more severe lupus than MRL controls, demonstrating a protective role for this gene in the pathogenesis of this condition [106]. Although numerous SNPs have been identified in the *TLR9* locus (chromosome 3p21.3), which falls into the SLE susceptibility region, there is very little correlation between these variants and the onset of SLE and again this is an area of major controversy within the literature. A number of these common SNPs (rs187084, rs5743836, rs352139, and rs352140) were investigated in a Hong Kong Chinese population [107], however although overrepresented in SLE showed no significant association. When the rs5743836 SNP was further analysed in Caucasian American individuals, again no functional association was identified with this polymorphism [108]. In contrast, studies in Brazilian patients replicated these results reporting this SNP as an SLE susceptibility factor (*P* = 0.045) [27]. Consistent with results seen by Tao et al. investigating TLR9, the exonic region rs352139A/G SNP has been mildly associated with SLE (*P* = 0.040), with genetic analysis in a Japanese population indicating that carrying the G allele of this polymorphism predisposes individuals to an increased risk of SLE through the downregulation of TLR9 expression levels in reporter gene assays [28]. In addition to this, when the rs352140C/T in exon 2, was examined using a family-based association in China it was also reported that this SNP was also mildly associated with disease susceptibility (*P* = 0.045) [29].

The divergent roles played by TLR7 and 9 in autoimmunity are reflected in the variation seen in *TLR7/9* polymorphisms and their subsequent effect on disease progression. TLR7 polymorphisms appear to increase expression of this gene leading to the enhanced recognition of autoantibodies culminating in an enhanced IFN signature thus predisposing these individuals to SLE [99]. On the other hand, TLR9 polymorphism associations, whilst controversial, particularly with respect to the different genetic backgrounds of the populations examined, suggest that *TLR9* SNPs downregulate its expression and in doing so increase disease susceptibility [107]. Although the exact mechanism for this is not yet known, it has been suggested that lower levels of TLR9 expression lead to defective T regulatory cell activation which contributes to the decrease in number and immunosuppressive function of these cells in the MRL model of murine lupus [106]. Despite some controversy surrounding individual *TLR7/9* SNPs in SLE, there is a growing body of evidence emerging to suggest that polymorphisms in these receptors play a role in genetic predisposition to this condition.

### 3.2. TLR Signalling Components

Activation of TLR7 and TLR9 by self nucleic acids and immune complexes has been demonstrated to contribute to the pathogenic production of IFN-*α* and proinflammatory cytokines such as TNF-*α*, IL-6 and IL-12 [109, 110]. A number of genes involved in type I IFN production and signalling have been linked to SLE [111–113]. Proteins directly activated downstream of TLR7 and TLR9, such as TRAF6 [12] and the IRF family of transcription factors [13], have known genetic association with SLE. In addition, proteins that negatively regulate TLR-induced activation of transcription factors IRF7 and NF-*κ*B such as A20, have also been shown to contribute to lupus susceptibility in a combination of either GWAS or candidate gene approaches [31, 33, 114]. 

#### 3.2.1. TRAF6

TNF-receptor-associated factor 6 (TRAF6) plays an important role in many signalling pathways that are important for immune regulation. TRAF6 was firstly identified in 1996 as a signal transducer in the NF-*κ*B pathway which associates with interleukin-1 receptor-associated kinase (IRAK) [115]. Recent studies have suggested that polymorphisms within *TRAF6* may be associated with the development of SLE, with SNPs in the *TRAF6* gene giving nominal signals of association with SLE in an extended family Swedish cohort [116]. A more recent study showed a direct correlation between *TRAF6* SNPs and SLE, supporting the notion that TRAF6 is potentially involved in the pathogenesis of autoimmune conditions [12]. In this study, fifteen SNPs across *TRAF6 *were evaluated in 7,490 SLE patients and 6,780 control subjects from different ancestries. Evidence of associations was detected in multiple SNPs, with rs5030437 and rs4755453 showing the strongest association [12]. 

#### 3.2.2. TREX1

*TREX1* encodes the most abundant 3′-5′ exonuclease in mammalian cells and has also been implicated in the cell death process, recognising and degrading genomic DNA and endogenous retroviral elements to minimize potential immune activation by persistent immunostimulatory DNA in the cytoplasm [117] (reviewed in [118]). Various genetic studies have identified a number of loss-of-function mutations in *TREX1* that give rise to SLE, familial chilblain lupus (FCL), or Aicardi-Gautieres syndrome (AGS), an autoimmune disorder that presents as early onset encephalopathy resulting in severe intellectual and physical handicap [119–122]. Functional studies into these loss-of-function mutants of *TREX1* demonstrate that they result in enhanced levels of immunostimulatory DNA resulting in enahnced type I IFN production. For example, *TREX1D18N* and *TREX1D200N* heterozygous mutants have been identified in FCL and AGS, respectively, and functionally are completely deficient at degrading dsDNA and demonstrate a lower rate of degradation of ssDNA than wild-type TREX1. The *TREX1R114H* homozygous mutation identified in AGS patients is found as a heterozygous mutation in SLE. As a homodimer *TREX1R114H* shows defects in its ability to degrade both ds- and ss-DNA, indicating that loss of function of TREX1 results in enhanced levels of immunostimulatory DNA which in turn results in enhanced levels of type I IFNs observed in both SLE and AGS [123, 124]. These findings clearly implicate TREX1 as an important endogenous DNA sensor that works to prevent inappropriate immune activation. 

#### 3.2.3. IRF5

Association of IRF5 genetic variants with SLE susceptibility has been first reported following a screening of genes involved in type I IFN production and response in Swedish, Icelandic, and Finnish patients with SLE [13]. Since then, the evidence of a link between IRF5 and SLE has been replicated in a number of case-control linkage studies in different populations [125–128] and GWAS analyses (reviewed in [129]). Association of IRF5 with SLE is complex, and a number of genetic studies have allowed defining risk, neutral, and protective haplotypes. Initially, 3 common polymorphisms in the *IRF5 *gene (SNPs rs2004640 in the 5′ UTR and rs10954213 in the 3′ UTR and a 30 nucleotides insertion in exon 6) [97] were proposed to alter the function or levels of IRF5 mRNA and proteins, thus explaining the association of risk alleles of these polymorphisms with SLE. A subsequent study by Sigurdsson et al. [130] identified two *IRF5* polymorphisms independently and strongly associated with SLE: a 5 bp CGGGG insertion located 64 base pairs upstream of IRF5 exon 1a (*P* = 4.6 × 10^−9^) and a SNP (rs10488631) downstream of the *IRF5* gene (*P* = 9.4 × 10^−10^). The presence of the insertion creates an additional binding site for the transcription factor Sp1, leading to increased transcription of IRF5 [130]. Interestingly, the CGGGG insertion is in linkage disequilibrium with SNPs rs2004640 and rs10954213, thus accounting for the association previously observed between these two SNPs and SLE. Interestingly, the CGGGG insertion in *IRF5* promoter has been associated with a number of autoimmune conditions, such as primary Sjögren's syndrome [131], Multiple sclerosis [132], inflammatory bowel disease and Crohn's disease [133], while the haplotype tagged by rs10488631 seems to be specific in conferring SLE susceptibility [130]. A recent study by Hedl and Abraham [134] has found that monocyte-derived cells from healthy individuals carrying the risk alleles of SNP rs2004640 and the CGGGG insertion secreted elevated levels of proinflammatory cytokines following stimulation with Nod2 and TLRs ligands, thus suggesting a correlation between IRF5 genetic variants and transcriptional activity. In keeping with this, it has been shown that patients carrying IRF5-risk haplotypes have increased levels of circulating IFN*α* in the serum compared to patients carrying neutral or protective haplotypes. Of note, such correlation was observed only in patients positive for either anti-dsDNA or anti-RBP autoantibodies [135], and the study was sub sequentially expanded to show that different classes of autoantibodies are linked to different *IRF5* haplotypes. Since autoantibodies can deliver self nucleic acids to endosomal TLRs [136], thus activating IRF5, the authors proposed that distinct classes of autoantibodies could activate specific IRF5 variants, leading to dysregulation of IFN*α* production and increased transcription of interferon-stimulated genes [137].

#### 3.2.4. IRF7

IRF7 is considered the master regulator of IFN*α* production downstream the antiviral TLRs [138], and polymorphisms in this gene could therefore be an ideal candidate for genetic susceptibility to SLE. Together with IRF5, IRF7 has been shown to be necessary for murine DCs-mediated production of IL-6 and IFN*α* induced by immune complexes isolated from SLE patients' sera, again indicating a central role for these transcription factors in the disease context [85]. SNPs in the genetic region spanning the *IRF7* gene (adjacent to the PHRF1 locus, also known as KIAA1542) have been identified, and different groups have attempted to associate common genetic variants at this site with SLE susceptibility. A GWAS in women affected by SLE has found a correlation between SNP rs4963128 in *KIAA1542* and lupus (*P* = 3 × 10^−10^). Since this SNP is in strong linkage disequilibrium with SNP rs702966 located within 0.6 kb of *IRF7*, it was thought that variability at this site could represent the signal deriving from *IRF7 *[78]. Association of these two SNPs with lupus susceptibility has been replicated in populations of different ancestries by Salloum et al. [139]. Interestingly, this study demonstrated a correlation between the risk alleles of these SNPs and increased levels of IFN*α*, but only in patients with autoantibodies. Similar to what has been suggested for IRF5, potential autoantibodies might cooperate with SLE-associated IRF7 variants through TLR activation, resulting in increased type I IFN production which leads to breaking of tolerance and the onset of disease. In keeping with this, SNP rs4963128 was correlated with nephritis and anti-Ro/anti-La autoantibodies in a Chinese population, although no association of this SNP with lupus susceptibility was observed in this genetic background [39]. To date, the only known functional polymorphism in *IRF7* is the nonsynonymous SNP rs1131665 which encodes a protein carrying a Q to R mutation at position 412 [30]. This variant has been shown to be associated with SLE patients of Asian and European American ancestry (*P* = 6.18 × 10^−6^), and functional analysis of the mutated protein revealed its enhanced transcriptional activation of an ISRE-dependent promoter. This is in keeping with the hypothesis that SLE-associated *IRF7* polymorphisms may lead to the expression of proteins with increased activity downstream of the TLRs, thus leading to overproduction of type I IFN characteristic of the disease.

#### 3.2.5. TNFAIP3

 Tumour necrosis factor *α*-induced protein 3 (TNFAIP3), the gene product of which is the ubiquitin editing protein A20, is an essential negative regulator of pathways regulating NF-*κ*B [140–142]. Recently TNFAIP3/A20 has been shown to interact with and negatively regulate IRF7, thus potentially explaining its molecular involvement in SLE [143]. Polymorphisms within the TNFAIP3 genomic locus, located at 6q23, have been associated with autoimmune disorders such as SLE [31, 35, 114, 144, 145] in Caucasian, Asian and Japanese populations. In particular, three independent SNPs in the TNFAIP3 gene (rs13192841, rs2230926 and rs6922466) are thought to be associated with SLE patients of European ancestry [31]. More recently the results of a meta-analysis of genome-wide association scans and replication in independent sets for *TNFAIP3* polymorphism and SLE showed another *TNFAIP3* SNP (rs2230926) to have an association with SLE [32]. This sample set contained 12,416 subjects with SLE from multiple ethnic groups and so suggested that this particular SNP may be conserved throughout diverse populations. In order to fully characterise the TNFAIP3 risk haplotype, fine mapping and genomic resequencing in ethnically diverse populations were carried out [146]. Results suggested a TT>A variant to be the most likely functional polymorphism responsible for the association between *TNFAIP3* and SLE in subjects of both European and Korean ancestry [146]. This variant displayed a reduced binding avidity for NF-*κ*B subunits. In addition, the haplotype carrying this variant resulted in reduced TNFAIP3 mRNA and A20 protein expression [146]. These findings underscore the crucial role of NF-*κ*B regulation in the pathogenesis of SLE. 

The TNFAIP3 interacting protein 1, (TNIP1), also known as ABIN1, interacts with TNFAIP3/A20 and promotes inhibition of NF-*κ*B activity [147, 148]. TNIP1 has also been shown to be associated with SLE in a wide range of ethnic groups. Two individual GWAS revealed association of TNIP1 intronic SNPs, rs7708392, and rs10036748, with SLE in both Caucasian and Chinese populations [35, 36]. Subsequently a study was carried out in a Japanese population which confirmed the association of TNIP1 rs7708392 with SLE [148]. Interestingly in this study, this SNP showed a tendency of stronger association with SLE patients with renal disorder than in all SLE patients. Overall these studies highlight the important role that both TNFAIP3 and TNIP1 play in genetic predisposition to autoimmune disorders such as SLE. 

### 3.3. Interferon Signalling Components

**Figure 2:**
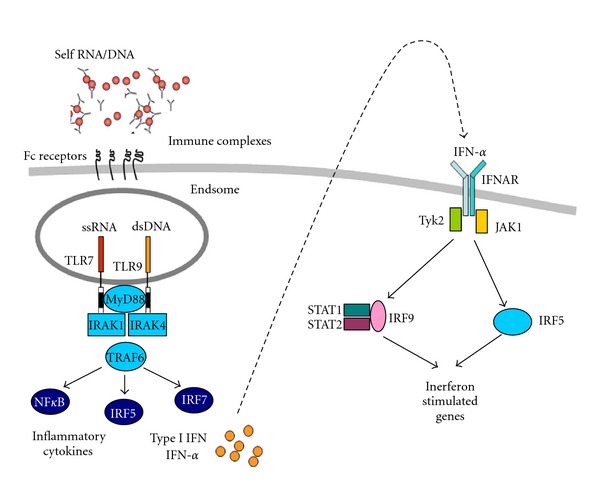
TLR induced IFN production and signalling in SLE. A brief outline of the signalling pathways involved in the production of type I IFNs in SLE. Activation of the transcription factors downstream of endosomal TLRs and Fc Receptors leads to the production of type I IFNs. These IFNs are secreted and further detected by IFN receptors, further activating interferon stimulated genes.

Serum levels of type I IFN correlate with disease activity and clinical manifestation [14], and interestingly lupus-like disorders can be induced during type I IFN therapy, again highlighting the pivotal role of these cytokines in disease development [149, 150]. Secreted type I IFN can then signal through the type I IFN receptor and kinases; tyrosine kinase 2 (TYK2) and janus kinase 1 (JAK1) [151]. Activation of the type I IFN receptor triggers phosphorylation of the transcription factors signal transducer and activator of transcription 1 and 2 (STAT1 and STAT2) and assembly of the interferon stimulated gene factor 3 (ISGF3) complex, which then translocates to the nucleus where it regulates production of IFN-stimulated genes necessary to establish the antiviral state (Figure 2) [152]. Polymorphisms in genes such as TYK2 and STAT4, involved in signalling downstream of the type I IFN receptor and a number of other cytokines, have been identified that might instead alter responses to type I IFN in SLE [37, 38, 41, 42, 153–159].

#### 3.3.1. STAT4

*STAT4*, the signal transducer and activator of transcription 4 gene, encodes a transcription factor that mediates the effect of several cytokines, including IL-12, the type I interferons, and IL-23 in T cells and monocytes [153]. Thus, STAT4 has a role in T-cell differentiation, monocyte activation, and IFN-*γ* production. STAT4 was confirmed in 2003 by Jacob et al. to play a key role in the pathogenesis of a lupus-like disease in mice [154]. They showed that loss of STAT4 led to accelerated renal disease and increased mortality. A number of genetic studies have identified *STAT4* SNPs with links to SLE in Caucasian populations for example, rs7582694 [37], rs7601754 and rs7574865 [38], and rs7582694 [155], in addition to rs7574865 and SLE in a Northern Han Chinese population [39]. Using transmission disequilibrium test analysis the rs7582694 SNP was found to have a strong association with SLE (*P* = 0.002, OR = 2.57) in a Finnish family cohort [37]. Using meta-analysis the SNPs rs7601754 and rs7574865 were found to have a significant association with SLE (*P* < 0.001) in populations of European and African origin [38]. Sigurdsson et al. (2008), in using a candidate gene study, also identified the SNP rs758294 as part of a common-risk haplotype for SLE (*P* = 1.7 × 10^−5^) in Swedish patients with SLE [155]. Li et al. (2011), using a candidate gene study in a Northern Han Chinese population, found a strong association between the SNP rs7574865 and SLE (*P* = 1.57 × 10^−6^) [39]. These SNPs are located within introns and are therefore suggested to play a role in the regulation of the expression level or splicing of the gene [155].

#### 3.3.2. TYK2

TYK2 binds to the type I IFN receptor (IFNAR), thus initiating the JAK-STAT signalling cascade, culminating in the transcription of further type I IFN and IFN inducible genes [156]. A number of SNPs in *TYK2 *have been recently reported to be associated with SLE in Caucasian populations, namely, rs280519, rs2304256, and rs12720270 [13, 41, 42]. The *TYK2* SNP rs280519 was found to be associated with SLE across a genome-wide association combined between a UK and Swedish cohort (*P* = 3.88 × 10^−8^) [41]. The *TYK2* SNP rs2304256, was found to be associated with SLE in a Scandinavian cohort (*P* = 5.6 × 10^−6^) [13], but not associated with SLE in a UK cohort [42], however this same UK study also found another *TYK2* SNP, rs12720270, associated with SLE that was not found within the Scandinavian cohort (*P* = 0.004). This SNP, rs12720270, however, was not found to be associated with SLE by Lee et al., when conducting meta-analysis on associations between SLE susceptibility and this SNP of *TYK2* [157]. The rs2304256 is located in exon 8, and the rare A allele of the SNP causes a substitution of Val to Phe at residue 362 in the Jak-homology 4 (JH4) region of TYK2. This region is important for the interaction of TYK2 with IFNAR1, its function [158], as well as for maintaining the expression of IFNAR1 on the cell surface [159], suggesting that this SNP may reduce the function of TYK2 and thus susceptibility to autoimmune diseases. 

## 4. Conclusion

Evidence from GWAS and candidate gene approaches have uncovered an array of genes that have functional consequences for how monocytes and macrophages respond to immune challenge during the course of disease. Many of these genes regulate either phagocytic, TLR, or IFN systems—three areas now well recognised to contribute to disease pathology. And as we become increasingly aware of the growing role of macrophages in disease pathology, it is interesting to note that cross-regulation of dendritic cells, the other major innate immune cell player in SLE pathology, by macrophages has an important role in driving disease. For example, *C1q* deficiency not only results in reduced uptake of immune complexes by macrophages and dendritic cells but it also is a negative regulator of IFN production by dendritic cells, thus its loss negatively impacts both macrophage and dendritic cell function in the context of disease pathology—exacerbating type I IFN production and contributing to a vicious cycle of reduced immune tolerance [160]. With respect to many of the genes discussed above, the functional relevance of their genetic variation has yet to be determined—do they contribute to pathogenic splice variants, altered transcript, or protein stability, or indeed introduce functional mutations that contribute to either over- or underactivation of the gene product? For others however, such as Trex1, not only is the molecular involvement of these variants in disease known, but research into their involvement in disease has uncovered novel functions for these proteins in innate immunity. However, where genetic associations uncovered have yet to conclusively demonstrate functional relevance for immune function in the context of SLE, we must be aware that many of the SNPs uncovered in SLE susceptibility regions may in fact have no true role in genetic susceptibility but instead, through linkage disequilibrium, act as a tag or marker for the real susceptibility gene. As researchers continue unravelling the functionality of genetic variability within SLE and translating these findings functionally to their contribution to immune dysregulation in SLE then we can undoubtedly expect this knowledge to contribute to greater insight into the molecular workings of disease. Already there are indications that certain SNPs appear to stratify with different disease manifestations and autoantibody profiles in SLE [161, 162], indicating the utility of screening to better inform and manage disease.

## Figures and Tables

**Table 1:** Polymorphisms of genes associated with SLE outlined in this review.

Category	Gene	SNP	Ethnicity
Immune complex recognition	C1q [8, 17, 18]		
	C1r [19]		
	C1s [20]	Rs292001 [21]	Turkish [17], Mexican [18]
	C2 [22]		
	C4 [8, 22–24]		

Nucleic acid recognition		rs3853839 [25]	Chinese, Japanese [25]
		rs179019 [26]	Japanese [26]
	TLR7 [25]	rs179010 [26]	
		rs179008 [27]	Brazilian [27]
		rs5743836 [27]	Brazilian [27]
	TLR9	rs352139 [28]	Japanese [28]
		rs352140 [29]	Chinese [29]
			Asian [30],
	IRF7	rs1131665 [30]	European American [30],
			African American [30]
		rs5030437 [12]	
	TRAF6	rs4755453 [12]	African American [12]
		rs540386 [12]	
		rs13192841 [31]	European [31]
		rs2230926 [31, 32]	European [31],
	TNFAIP3	rs6922466 [31]	Chinese Han [33]
		rs5029939 [34]	European [31]
		rs7708392 [35, 36]	Caucasian, Chinese [35, 36]
	TNIP1	rs10036748 [35, 36]	Japanese [26]
			Caucasian, Chinese [35, 36]

Interferon signalling		rs7582694 [37]	Caucasian [37]
	STAT4	rs7601754 [38]	Caucasian [38]
		rs7574865 [38]	Caucasian [38],
			Northern Han Chinese [39]
		rs7582694 [40]	Caucasian [40]
	TYK2	rs280519 [41]	UK, Swedish [41]
		rs2304256 [13]	Scandinavian [13]
	IRF5	rs12720270 [42]	UK [42]
		rs10488631 [43]	
